# pH-Responsive PEGylated Niosomal Nanoparticles as an Active-Targeting Cyclophosphamide Delivery System for Gastric Cancer Therapy

**DOI:** 10.3390/molecules27175418

**Published:** 2022-08-24

**Authors:** Farnaz Khodabakhsh, Mahsa Bourbour, Mohammad Tavakkoli Yaraki, Saina Bazzazan, Haleh Bakhshandeh, Reza Ahangari Cohan, Yen Nee Tan

**Affiliations:** 1 Department of Genetics and Advanced Medical Technology, Medical Biotechnology Research Center, Faculty of Medicine, AJA University of Medical Sciences, Tehran 1411718541, Iran; 2 Department of Biotechnology, Alzahra University, Tehran 1993893973, Iran; 3 School of Natural Sciences, Macquarie University, Sydney, NSW 2109, Australia; 4 Department of Community Medicine, Mashhad Branch, Islamic Azad University, Mashhad 9187147578, Iran; 5 Department of Nanobiotechnology, New Technologies Research Group, Pasteur Institute of Iran, Tehran 1316943551, Iran; 6 Faculty of Science, Agriculture and Engineering, Newcastle University, Newcastle Upon Tyne NE1 7RU, UK; 7 Newcastle Research and Innovation Institute, Newcastle University in Singapore, Singapore 609607, Singapore

**Keywords:** gastric cancer, Cyclophosphamide, Niosome, PEGylation, optimization, drug delivery

## Abstract

A PEGylated niosomal formulation of cyclophosphamide (Nio-Cyclo-PEG) was prepared using a central composite design and characterized in terms of drug loading, size distribution, and average size. The stability of formulations was also studied at different conditions. In vitro cytotoxicity of drug delivery formulations was assessed on gastric cancer cells using MTT assay. The mechanism of cytotoxicity was studied at the transcriptional level by real-time PCR on Caspase3, Caspase9, CyclinD, CyclinE, MMP-2, and MMP-9 genes, while apoptosis was investigated with flow cytometry. The anti-metastatic property was evaluated using the scratch method. Propidium iodide staining was used to study the cell cycle. The results indicated that the as-designed nanocarrier exhibited a controlled drug release pattern with improved nanoparticle stability. It was found that the living cancer cells treated with Nio-Cyclo-PEG showed a significant decrease in number when compared with the niosomal carrier without PEG (Nio-Cyclo) and free drug (Cyclo). Moreover, the drug-loaded nanocarrier induced planned death (apoptosis) in the cancer cells through the regulation of Caspase3, Caspase9, CyclinD, CyclinE, MMP-9, and MMP-2 gene expression, indicating that the Nio-Cyclo-PEG formulation could significantly inhibit the cell cycle at the sub G1 phase as well as prevent the migration of cancer cells. In conclusion, Nio-Cyclo-PEG as developed in this study could serve as an active-targeting drug delivery nanocarriers for gastric cancer therapy with high efficacy and minimal side effects on healthy tissues/cells.

## 1. Introduction

One of the most practical roles of nanotechnology in pharmaceuticals is to improve the physicochemical properties of drug delivery carriers [1,2,3]. Conventional formulations distribute drugs to the whole body nonspecifically, resulting in severe bystander effects. On the other hand, nanotechnology provides a facility to design smart nanocarriers, which not only could deliver specific drugs or biomolecules to the target site for disease treatment (e.g., cancer cells) but also have the benefits of increasing drug solubility, protecting drug compounds against harmful enzymes, controlling drug release into the circulatory system, and so on [4,5]. The biological and physicochemical properties of nanocarriers for drug delivery can be optimized using different formulations and surface modification, which allow them to penetrate more easily into the target cells and protect the drug from enzymatic degradation [4,5]. The shape, size, and composition of nanocarriers [6] also can be tuned to control the drug entrapment and release profiles, as well as improve their stability and targeting ability [4,7].

Nanocarriers for drug delivery can be categorized into two main groups: inorganic and organic. Since inorganic nanocarriers usually contain metal (e.g., metal–organic frameworks, porous metal oxides), they mostly suffer from low biocompatibility [8,9]. Among organic nanocarriers, liposomes and niosomes are highly efficient for therapeutic delivery purposes. About 10 years after Bingham’s discovery of liposomes, it was discovered that fatty acids and nonionic surfactants can form vesicles called niosomes that are more cost-effective and stable than liposomal nanocarriers for drug delivery [10,11]. Aside from good biocompatibility, the niosome nanocarrier is cost-effectiveness and can increase the half-life of the drug. Currently, niosomes are used as unique carriers for the encapsulation of both hydrophobic and hydrophilic drugs [12]. As with liposomal systems, surface modifications can be applied to improve the specificity and efficiency of niosomes. Polyethylene glycol (PEG), which is a non-ionic and hydrophilic polymer, is one of the most common biocompatible polymers for the surface modification of niosomes. PEG-modified niosomes are indistinguishable from the reticuloendothelial system and therefore enhance the in vivo half-life of the nanocarrier for drug delivery [13,14,15]. In addition, the use of biofunctionalized niosomes in chemotherapy can improve active-targeting delivery of drug to the tumour site [16,17,18,19,20]. Moreover, they penetrate better into the solid tumours and provide the needed therapeutic dosage/concentration for the inhibition of tumour growth.

Among the most common cancers in the world, gastric cancer ranks fifth in incidence and fourth in mortality. Both genetic and environmental factors are involved in this type of cancer [21,22]. Gastric cancer is associated with an uncontrolled growth of epithelial cells, and the initial symptoms include heartburn, upper abdominal pain, nausea, and anorexia [23,24]. One of the most powerful chemotherapeutic drugs is cyclophosphamide, which inhibits DNA replication as well as cell proliferation and survival [25,26]. Once cyclophosphamide reaches the body, it will be converted by liver enzymes into active metabolites, namely, phosphoramide mustard and acrolein. Phosphoramide mustard has anticancer properties, while acrolein exhibits cytotoxicity-like apoptosis and necrosis. In general, the mechanism of action of cyclophosphamide is through the irreversible binding between or within two strands of DNA at the guanine N-7 positions that leads to cell apoptosis [26]. Although many studies have examined the effects of cyclophosphamide on gastric cancer and confirmed its function in inhibiting cancer cells, the effect of cyclophosphamide loaded on PEGylated niosomes has not yet been evaluated. To the best of our knowledge, niosome-based drug delivery system has rarely been used to treat gastric cancer [27]. Therefore, designing a pH-responsive niosomal formulation to deliver cyclophosphamide into gastric cancer tissues is of great importance.

In this study, a PEGylated niosome-based formulation of cyclophosphamide was developed using a central composite design (CCD) and was comprehensively characterized in terms of its drug loading, size distribution, morphology, and release profile. The physical stability of niosomal formulations was studied under different storage conditions. In addition, we also carefully examined the biological properties of the formulated niosome and understood how it works on the gastric cancer cells through various cellular tests. We also studied the cytotoxicity of drug-loaded niosome on gastric cancer cells and its mechanism of action at the transcriptional level studied and compared these with free cyclophosphamide in solution. Finally, the function of this novel formulation on metastasis and cell cycle was evaluated, which suggested the effectiveness of this nanoengineered drug delivery system for gastric cancer treatment.

## 2. Result and Discussion

### 2.1. Optimization Studies of Cyclophosphamide-Loaded Niosomes

**Scheme 1:**
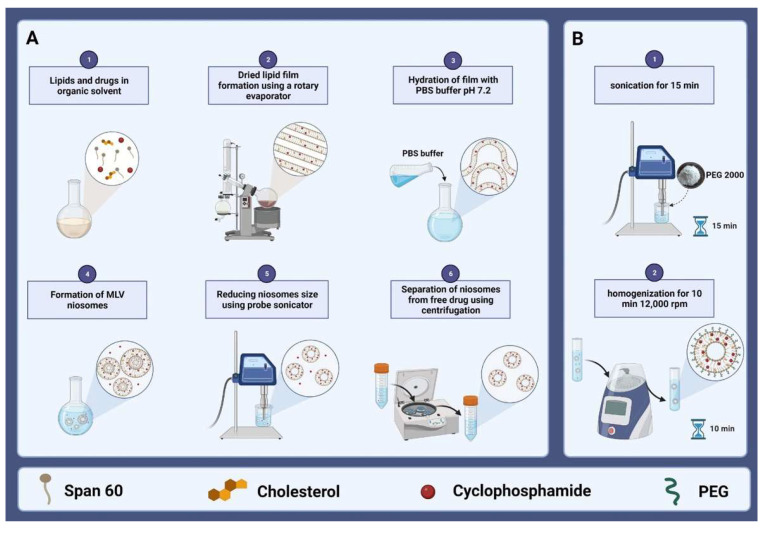
The preparation (**A**) and PEG functionalization (**B**) of cyclophosphamide-loaded niosomes via thin-layer hydration.

As shown in Scheme 1, thin-layer hydration was used to prepare and functionalize the cyclophosphamide-loaded niosomes (Nio-Cyclo). The effects of three independent variables, the lipid-to-drug molar ratio, surfactant-to-cholesterol molar ratio, and surfactant type, on drug entrapment efficiency (EE), nanoparticle size, and size distribution (polydispersity index: PDI) were investigated to reach the optimal formulation of PEG functionalized cyclophosphamide-loaded niosomes (Nio-Cyclo-PEG). Several important parameters (independent variables) of forming the cyclophosphamide-loaded niosomes include (1) molar ratios of surfactants to cholesterol and, (2) molar ratios of lipid to drug as well as (3) type of surfactant such as Span 20 or Span 60. These parameters were investigated carefully to optimize the nanocarriers’ drug delivery responses, such as entrapment efficiency (%), nanoparticle size, PDI, and loading capacity (%). The observed responses related to the abovementioned three independent variables (lipid:drug molar ratio, surfactant:cholesterol molar ratio, and surfactant type) were compared with the predicted values as obtained by the mathematical model (i.e., quadratic polynomial model) in the response surface methodology (RSM) for formulation optimization. The models were assessed in term of statistically significant coefficient, and Table 1 shows the response values after the experiments.

#### 2.1.1. Analysis of Particle Size

The particle sizes of niosomal drug carriers resulting from different formulations are shown in Table 1. A quadratic model was fitted on particle size data with statistical significance set at *p* value < 0.0001. ANOVA (Table 2) results showed that all three independent variables affect particle size (*p* value < 0.05). The summary of results for quadratic polynomial model is given in Table 3. In more detail, the lipid: drug and surfactant: cholesterol molar ratios possess positive and negative effects, respectively. Important factors in the formation of bilayer vesicles are the hydrophilic–lipophilic balance (HLB) of non-ionic surfactants, critical packing parameters and the structure of constituents [28]. With an increase in the amount of cholesterol, the average diameter of the two layers of the niosome structure also increases, and competition between the drug and cholesterol follows. Therefore, an excessive increase in cholesterol in niosome structures tends to deposit in two layers of niosomes, which can lead to drug removal from the structure [29].

**Figure 1:**
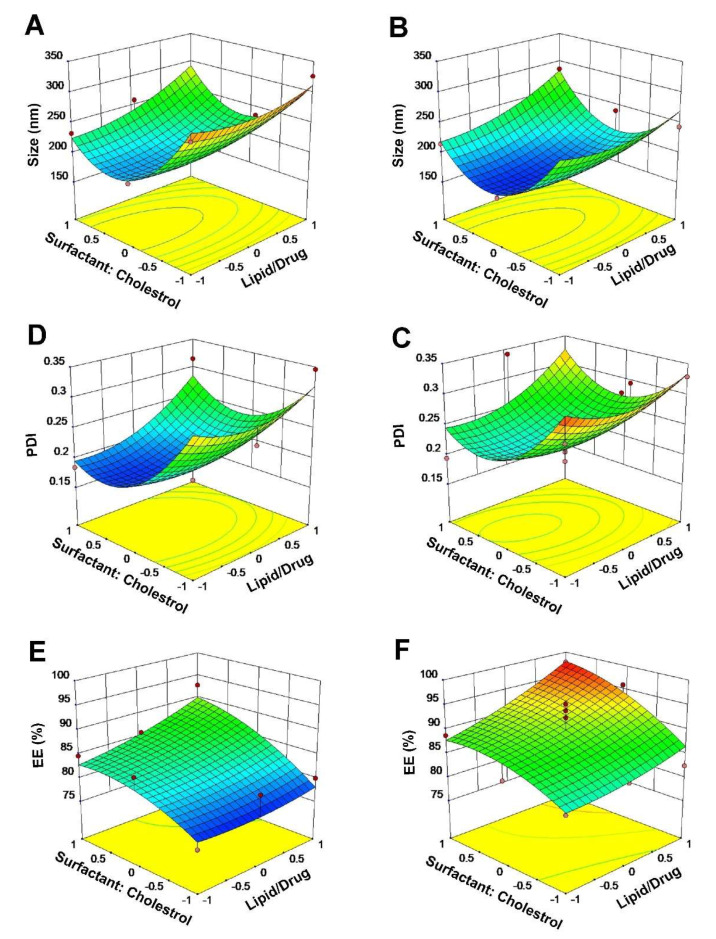
Effects of different variables on size of niosomes, polydispersity index of niosomes and entrapment efficiency of drug by niosomes when Span 20 (**A**,**C**,**E**) and Span 60 (**B**,**D**,**F**) were used in the formulation.

The 3D plots in Figure 1A,B illustrate the simultaneous effects of independent variables on the particle size response and the effects of two individual variables (i.e., surfactant:cholesterol and lipid:drug molar ratios for both surfactants) on the response one at a time. These results show that the lipid:drug molar ratio has a positive effect on particle size. However, the particle size decreases with increasing the surfactant: cholesterol molar ratio. This phenomenon was also observed for the niosomes formulated using two different surfactants, i.e., Span 20 (Figure 1A) and Span 60 (Figure 1B).

#### 2.1.2. Polydispersity Index Analysis

According to Table 2, only independent variable B (i.e., surfactant: cholesterol molar ratio) had a significant effect on PDI, with a *p* value < 0.05. A quadratic model was fitted to the response data. Table 3 presents the regression equation for PDI. As can be seen in Figure 1C,D, independent variable B has a negative effect on PDI, meaning that for both niosomes, containing Span 20 and Span 60, PDI decreases as B increases.

#### 2.1.3. Entrapment Efficiency (EE%) Analysis

As shown in Table 2, all three independent variables (A–C) have significant effects on EE% (*p* value < 0.05). The minimum and maximum entrapment efficiency were 75.42% and 97.74% for the F5 and F15 formulations, respectively (Table 1). The fitted model was statistically significant (*p* value < 0.05). Table 3 also displays the regression equation for EE%. It can be concluded that entrapment efficiency is proportional to the independent variables A and B as revealed by the equation. In other words, the entrapment efficiency increases with an increase in each variable (Figure 1E,F). For HLB > 6, cholesterol must be added to the surfactant until a bilayer vesicle form. The presence of cholesterol in the structure of nano-sized niosomes is essential because it can inhibit the accumulation of surfactants, reduce drug leakage, and reduce particle size ^28^.

#### 2.1.4. Accuracy and Validity of the Model

Table 4 shows the regression analysis results for EE, particle size, and PDI. There is a reasonable agreement between R^2^ and adjusted R^2^ that represents the validity of model to predict the responses. In addition, adequate precision for all responses is greater than 4, indicating an adequate signal-to-noise ratio. Therefore, this ratio implies the suitability of the model to navigate the design space [30].

#### 2.1.5. Data Optimization

After applying the limits on particle size and entrapment efficiency percentage, this optimization study proposed an optimal formulation with a good desirability index. This index is a kind of multi-criterion optimization and is used when one response must be at the minimum and the other must be at the maximum to have the optimal formulation [31]. Given the minimum particle size, narrow polydispersity, and maximum entrapment efficiency, the optimal condition was determined. A good desirability index (desirability = 0.978) was achieved for the optimized formulation make up of lipid:drug molar ratio of 18.4 and surfactant (Span 60): cholesterol molar ratio of 1.288. The predicted values for particle size, PDI, and EE of the optimized formulation was 171.65 nm, 0.18 and 90.33%, respectively, which were very close to the experimentally observed values (i.e., particle size: 188.1 nm, PDI: 0.17, and entrapment efficiency: 92.58%). Table 5 indicates the correlations between the experimental data and the predicted values for the three responses for different samples. There are good agreements between the observed and predicted values that confirm that the experimental data are well described by the selected quadratic model.

In the current study, 5% weight to volume (*w*/*v*) of PEG 2000 was added to the optimized formulation to achieve smaller niosomes with improved stability, less aggregation, and higher entrapment efficiency (Table 5). According to the results, the optimum niosomal formula modified with PEG (Nio-Cyclo-PEG) exhibited a higher drug entrapment (94.87%), smaller diameter (170.6 nm), smaller PDI (0.145), and lower drug release than the optimum formulation without PEG (Nio-Cyclo). Similar results were also observed in previous works [13,32], where PEGylation provides in vivo and in vitro stability to the drug nanocarriers formulations [13,33]. It was also reported that the use of PEGylation in niosome structures lead to the improved stability, increased drug encapsulation, reduced particle size , and controlled drug release [13].

### 2.2. Size Distribution and Morphological Characterization

**Figure 2:**
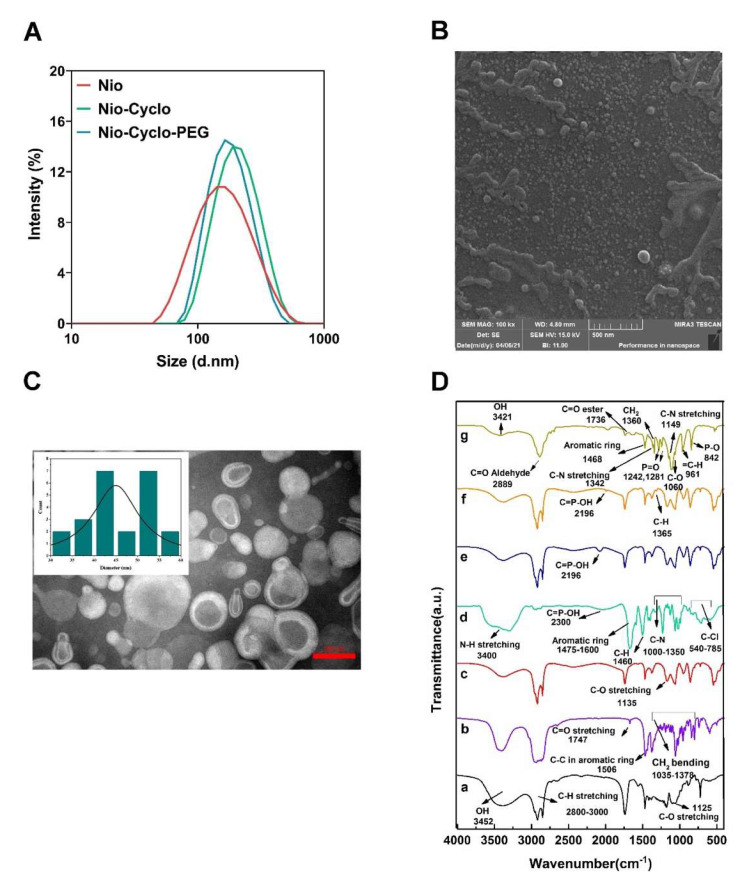
(**A**) Particle size distribution of Niosome (Nio), Cyclophosphamide-loaded niosome (Nio-Cyclo), and PEGylated Cyclophosphamide-loaded niosome (Nio-Cyclo-PEG) formulations obtained by DLS. (**B**) Morphological assessment of the best Nio-Cyclo formulation using scanning electron microscopy (SEM), and (**C**) transmission electron microscopy (TEM); (**D**) FTIR spectra of (**a**) Span60, (**b**) Cholesterol, (**C**) Niosome, (**d**) Cyclophosphamide, (**e**) Nio-Cyclo, (**f**) Nio-Cyclo-PEG, and (**g**) PEG 2000.

Size distribution and morphological studies of the best formulation were investigated using scanning electron microscopy (SEM), dynamic light scattering (DLS), and transmission electron microscopy (TEM). As shown in the SEM image (Figure 2B), Nio-Cyclo has a uniform semi-spherical shape with smooth surface characteristics. SEM confirmed that the niosomes have uniform spherical morphology. The diameter of the Nio-Cyclo particles as observed on SEM was smaller than that obtained by DLS (Figure 2A). This difference might be due to the drying of samples prepared for SEM imaging. Typically, SEM images provide the morphological information of nanoparticles in a dried form (measuring the exact diameter of each nanoparticle), while DLS shows the hydrodynamic diameter that includes the size of core and all the adsorbed molecules (water and ions) on the surface [34]. The transmission electron microscopy (TEM) images in Figure 2C show that the optimized Nio-Cyclo are spherical in shape with structurally rigid boundaries. Additionally, the zeta potential of Nio-Cyclo (Figure S1A, Supplementary Materials) and Nio-Cyclo-PEG (Figure S1B, Supplementary Materials ) were found to be −24.7 mV, and −15.0 mV, respectively.

### 2.3. Fourier Transform Infrared Spectroscopy (FTIR) Spectroscopy Analysis

FTIR analysis was performed to confirm the presence of cyclophosphamide in the niosomal formulations for drug delivery. The FTIR spectrum of Span 60 in Figure 2D(a) shows several specific peaks that include O–H stretching at 3452 cm^−1^, C–O stretching at 1125 cm^−1^, and the C–H stretching in the region of 2800–3000 cm^−1^. Figure 2D(b) shows the FTIR spectrum of cholesterol where the spectrum’s bands can be assigned to the C=O stretching at 1747 cm^−1^, C–C stretching in the aromatic ring at 1506 cm^−1^, OH stretching at 3452 cm^−1^, C=C stretching at 1674 cm^−1^, C–H stretching in the region of 2800–3000 cm^−1^, and CH_2_ bending and CH_2_ deformation in the region of 1035–1378 cm^−1^. The FTIR pattern for blank niosome (Figure 2D(c) displays various characteristic peaks of components in the range of 3400–1125 cm^−1^. The band observed at 3452 cm^−1^ was assigned to cholesterol and Span 60 (OH stretching), while CH stretch that occurred in the region of 2800–3000 cm^−1^ belonged to cholesterol and Span 60. Figure 2D displays the FTIR spectrum of cyclophosphamide that can be assigned to N–H stretching at 3400 cm^−1^, C=P–OH bond in 2300 cm^−1^, C-CL bond in 540–785 cm^−1^, and C–N in the region of 1000–1036 cm^−1^. The C=C stretching (at 1674 cm^−1^) bands of cholesterol vanished in the case of niosomes, confirming its entrapment in the lipid bilayer of the structure. As shown in Figure 2D(c) the C–H stretching peak (1460 cm^−1^) and C–O stretching peak (1125 cm^−1^) in the cholesterol and drugs shifted to the 1464 cm^−1^ and 1169 cm^−1^, respectively. The FTIR spectra of the niosomes, which could be due to the changes in forces after interaction of molecules of drug with cholesterol and span 60, further confirm the presence of drug molecules and cholesterol inside the shell (lipid bilayer) of the drug-loaded niosomes. These prominent peaks of drug molecule do not exist in the drug-loaded niosomes, which indicates the successful entrapment of drug by the niosomal nanocarriers. As cyclophosphamide became trapped in the niosome, a peak appeared at 2196 cm^−1^ that was related to the C=P–OH (Figure 2D(e). Additionally, the FTIR spectrum obtained from the PEGylated niosomal cyclophosphamide (Nio-Cyclo-PEG) depicted a tiny peak that was related to the CH_2_ of PEG’s chain, indicating the presence of PEG in the formulation (Figure 2D(f).

### 2.4. Drug Release and Kinetic Modelling

**Figure 3:**
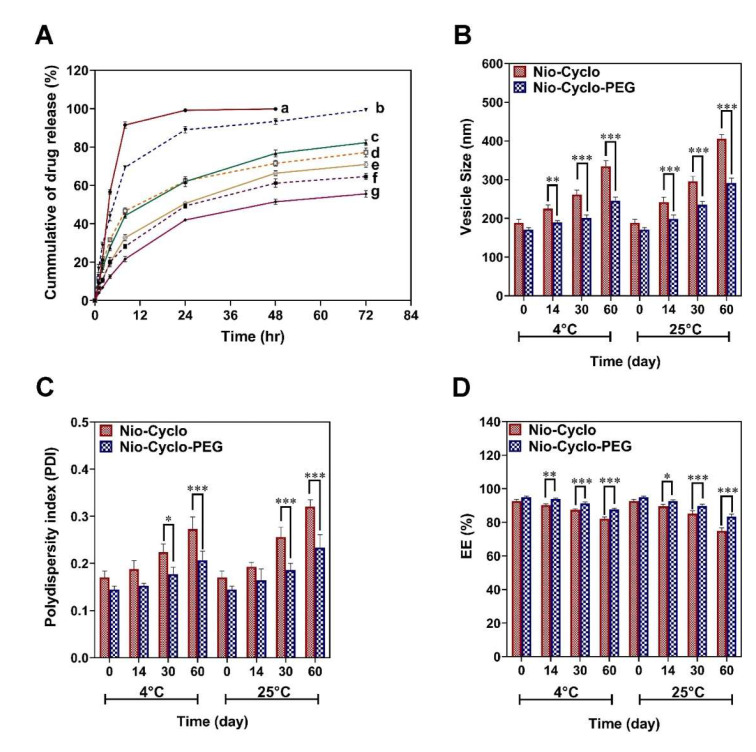
(**A**) In vitro drug release profile of cyclophosphamide from the Nio-Cyclo and Nio-Cyclo-PEG nanocarriers at 37°C. (**a**) Free Cyclophosphamide (Cyclo), (**b**) Nio-Cyclo at pH 1.2, (**c**) Nio-Cyclo at pH 5.4, (**d**) Nio-Cyclo-PEG at pH 1.2, (**e**) Nio-Cyclo-PEG at pH 5.4, (**f**) Nio-Cyclo at pH 7.4, and (**e**) Nio-Cyclo-PEG at pH 7.4; (**B**–**D**) Stability of optimum Nio-Cyclo and Nio-Cyclo-PEG formulations after 2 months of storage at 4 and 25 °C. Data are represented as Mean ± SD from three independent experiments (*: *p* < 0.05, **: *p* < 0.01 and ***: *p* < 0.001).

An essential property of a delivery system is the control release ability of drug at the tumour site. In this study, the drug release profile of Nio-Cyclo and Nio-Cyclo-PEG was investigated for 72 h in PBS-SDS medium under a controlled condition with constant stirring at 37 °C. The release of cyclophosphamide from Nio-Cyclo and Nio-Cyclo-PEG nanocarriers were further investigated at physiological (~7.4), pathological cancerous (~5.4) pH conditions and gastric fluid pH condition (~1.2). As shown in Figure 3A, the drug release was pH dependent. A slow release of drug at physiological pH 7.4 was observed within 72 h, while the release profile at acidic pH (5.4) was faster, which is one of the prerequisites for the transport of anticancer drugs like cyclophosphamide to the acidic surroundings of cancerous tissues. However, according to the observed results, the fastest drug release is present at pH 1.2, which is associated with the pH of gastric fluid.

These results clearly demonstrate the considerable role of niosomal formulation in preventing the burst release of drug at pH of 7.4 (physiological condition) [35]. Moreover, an electrostatic interaction between the drug and the surfactant is necessary in cases where the drug is in its ionization state under physiological pH [36]. Nio-Cyclo and Nio-Cyclo-PEG formulations showed a biphasic release profile with an initial burst release in the first 8 h, which can be attributed to the drug’s diffusion from the outer layers of niosomes [37]. Following the burst release, gradual releases were observed of 35% and 25% for the Nio-Cyclo and Nio-Cyclo-PEG nanocarriers, respectively. When the drug was released within 72 h, a stationary phase was observed for all conditions. This phenomenon can be explained by the barriers in the hydrophobic drug’ passing through the hydrophobic vesicular membrane, which causes a slower release [37]. Decreasing the pH resulted in a remarkable release of cyclophosphamide within 72 h (i.e., 82% and 71% for Nio-Cyclo and Nio-Cyclo-PEG, respectively). This could be attributed to the swelling of niosomes at acidic pH, and in turn their breaking, which is a normal behaviour of the niosomes under such conditions [38,39]. Another possible mechanism for this behaviour could be the faster hydrolysis of surfactants at acidic pH, leading to a burst release of drug molecules at lower pH [40]. Moreover, the rapid release of drugs at acidic pH or higher temperature can be due to the solubilization of aggregated drugs and the loosening of the Nio and/or Nio-PEG membranes [41]. While 92% of free drug was released within the first 8 h from the dialysis bag, Nio-Cyclo-PEG exhibited a slower release profile than the Nio-Cyclo nanocarriers, showing its potential application for the controlled release delivery of cyclophosphamide to the solid tumours [42]. In short, this drug release study clearly illustrated that the Nio-Cyclo-PEG formulation can improve the availability of drug in a more extended period, while the cytotoxicity effects are reduced, which results were in agreement with the previous studies [43].

The release kinetics of cyclophosphamide drug from the Nio-Cyclo and Nio-Cyclo-PEG nanocarriers are presented in Table 6. The free cyclophosphamide (Cyclo) follows the first-order kinetic model. According to this first-order model, the drug release rate depends on its concentration. The best-fitted release kinetic model for cyclophosphamide from the Nio-Cyclo and Nio-Cyclo-PEG nanocarriers were determined from the correlation coefficients of the highest values. According to the results, the Korsmeyer–Peppa model could reliably estimate the drug release behaviour of nanocarriers near the physiological and cancerous pH conditions. The obtained n values for each sample at different pH (0.43 < *n* < 0.85) indicate the anomalous transport mechanism (i.e., the combination of Fickian diffusion and erosion) for the release of the drug from the niosomal formulations [44]. The diffusion coefficient of Korsmeyer–Peppa’s model was found to be decreased when the Nio-Cyclo and Nio-Cyclo-PEG nanocarriers were made in contact with cancerous cells under acidic pH instead of the physiological pH. In fact, with a decreased drug release rate, it became less controlled by Fick’s law. In addition, the percentages of release at pH 1.2 for empty niosomes were calculated as 69.4%, 89%, and 99.3% in the time intervals of 8, 24, and 72 h, respectively. On the other hand, lower percentages of drug release were observed for the PEGylated niosomes under the same period of time, i.e., 8 h (46.7%), 24 h (62.3%) and 72 h (77.2%). It should be noted that the obtained kinetics for the empty niosome and PEGylated niosome follow the first-order kinetic and Korsmeyer–Peppas models, respectively.

### 2.5. Physical Stability Examination

A stable niosomal formulation must exhibit uniform nanoparticle size and a constant level of entrapped drugs with no precipitation [45]. Thus, the particle size, PDI, and percentage of cyclophosphamide remaining in the optimized Nio-Cyclo and Nio-Cyclo-PEG formulations were measured for 60 days under the storage conditions of 4 ± 2 °C and 25 ± 2 °C, respectively. The comparisons of the experimental data in Figure 3 indicated that the particle size (Figure 3B), PDI (Figure 3C), and entrapment efficiency (Figure 3D) of Nio-Cyclo-PEG formulation stored at both temperatures were more stable than the Nio-Cyclo formulation. A significant difference (*p* < 0.05) was observed between the Nio-Cyclo and Nio-Cyclo-PEG formulations stored at 4 and 25 °C in terms of their particle size, PDI, and EE%. The presence of PEG in the formulation exhibited promising effects on the encapsulation efficiency, particle size, and PDI after 60 days of storage. This indicates that the PEGylation of niosomal formulations could minimize the drawbacks associated with niosomal instability including fusion, aggregation, and drug leakage [41].

Despite the physical stability of Nio-Cyclo and Nio-Cyclo-PEG nanocarriers during the storage period, it was observed that the entrapped drug leaked gradually from the niosomal formulations, especially at 25 °C storage conditions (Figure S2E,F, Supplementary Materials). As a result, after two months of storage, a significant difference was detected in the entrapment efficiency of niosomal formulations at both storage temperatures (*p* < 0.05). There was a direct relationship between the entrapment efficiency and the storage time. Increasing the temperature also resulted in a higher rate of leakage. The greater leakage at higher temperatures for both samples (Nio-Cyclo and Nio-Cyclo-PEG formulations) can be assigned to the high fluidity of the lipid bilayer at these temperatures [46]. This high fluidity facilitates the nanocarrier fusion. During fusion, some unstable and large niosomal nanocarriers rupture, leading to drug leakage. Additionally, the fatty acid chain of the used surfactants adopts an irregular configuration at high temperatures. Thus, the rate of diffusion across the bilayer membrane increases with decreasing bilayer thickness [47]. Moreover, due to the fusion and aggregation of niosomal nanocarriers [48], the mean particle size and PDI may increase upon storage. The PDI and particle size enlargement was greater at 25 °C (Figure S2A–D) than the 4 °C. This effect may be attributed to a lower permeability and mobility of niosomal nanocarriers at 4 °C [49], whereas niosomes stored at 25 °C tend to fuse and form larger vesicles [14]. These results indicated that both the Nio-Cyclo and Nio-Cyclo-PEG formulations are more stable to be stored at 4 °C than that at 25 °C.

### 2.6. Cytotoxicity Study

**Figure 4:**
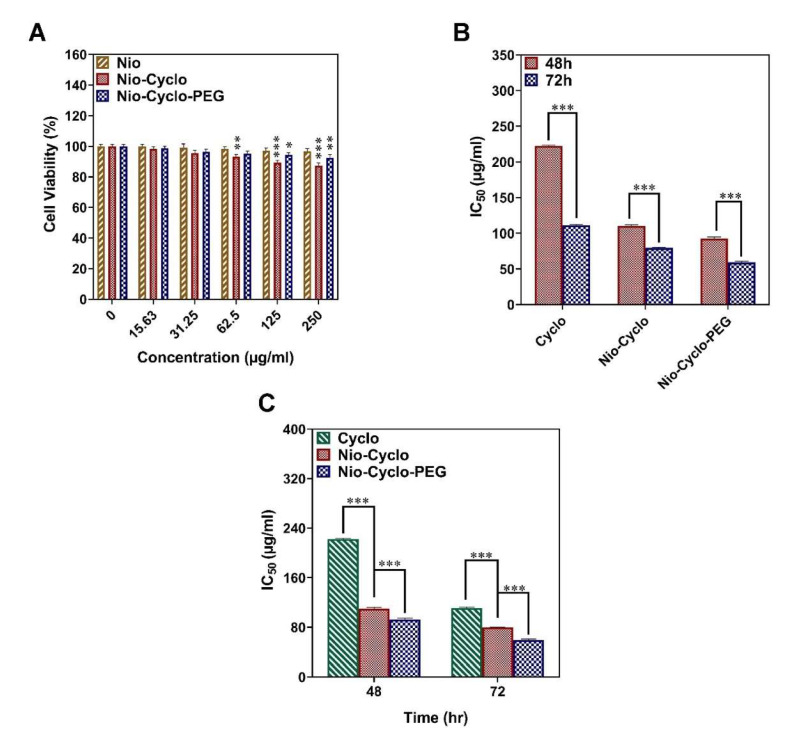
(**A**) Cell viability after treating the HFF cells with different concentrations of niosomal formulations. (**B**) The effect of time on IC_50_ for each sample against AGS cells. (**C**) Comparison of IC_50_ in groups at 48 and 72 h tested on the AGS cells. Data are represented as Mean ± SD from three independent experiments (*: *p* < 0.05, **: *p* < 0.01 and ***: *p* < 0.001).

Cytotoxicity study was carried out on the Cyclo, Nio-Cyclo and Nio-Cyclo-PEG on the HFF and AGS cell lines. The results of MTT assay indicated that with increasing concentration of drug loaded nanocarriers, the viability of treated HFF cells with Nio-Cyclo and Nio-Cyclo-PEG decreases (Figure 4A). In particular, a significant decrease in cell viability was observed under two tested concentrations, i.e., 125 and 250 µg/mL, for the Nio-Cyclo and Nio-Cyclo-PEG nanocarriers, respectively. However, increasing the concentration of drug-free niosomes did not have any significant effect on the viability of HFF cells, which confirms the non-toxicity of empty niosomes on the cells. Figure 4B shows the effect of incubation time of drug-loaded nanocarriers with AGS cells on IC_50_. As can be seen in Figure 4B, IC_50_ in all three samples (Cyclo, Nio-Cyclo and Nio-Cyclo-PEG) decreases significantly with increasing time from 48 h to 72 h on AGS cells. The IC_50_ values on AGS cells at 48 h were 222.26 ± 1.26%, 110.08 ± 1.89%, and 92.55 ± 2.41% for the Cyclo, Nio-Cyclo, and Nio-Cyclo-PEG, respectively; IC_50_ values obtained at 72 h of incubation with AGS cells showed similar trends for the respective drug formulations (i.e., 111.04 ± 1.49% (Cyclo), 79.50 ± 0.92% (Nio-Cyclo), and 59.25 ± 1.82 (Nio-Cyclo-PEG).

In addition, as shown in Figure 4C, IC_50_ for the drug-containing niosomes (Nio-Cyclo) was significantly reduced compared with the free drug (Cyclo) at both drug treatment durations (48 h and 72 h) on AGS cells. In particular, Nio-Cyclo-PEG shows a significant reduction in IC_50_ compared with the other samples (Cyclo, Nio-Cyclo), indicating that a low concentration of drug is effective and thus will have lower toxicity when administered to the patient. In general, Figure 4C shows that over time, Nio-Cyclo-PEG exhibited lower IC_50_ than Nio-Cyclo or Cyclo on AGS cells. Studies have shown that niosome could effectively deliver chemotherapy drugs to a variety of cancer cells [50,51], and a niosomal structure with PEG did not cause cell toxicity [52,53].

### 2.7. Gene Expression Analysis

**Figure 5:**
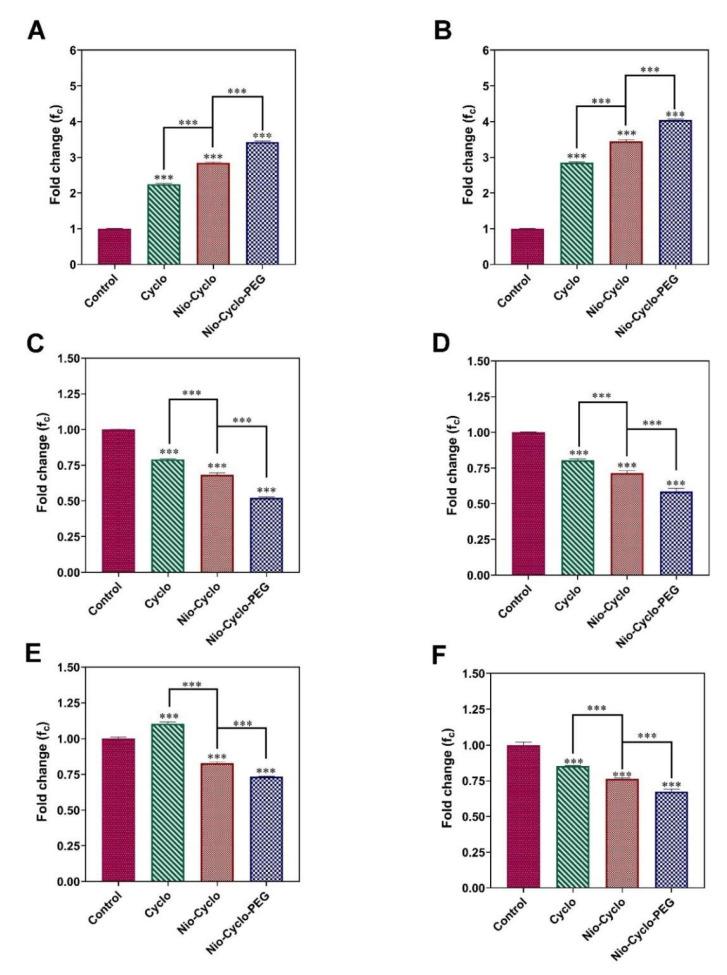
The expression profiles of (**A**) *Caspase 3*, (**B**) *Caspase 9*, (**C**) *Cyclin D*, (**D**) *Cyclin E*, € *MMP-2*, and (**F**) *MMP-9* in AGS cells after treatment with different niosomal formulations; Data are represented as Mean ± SD from three independent experiments (***: *p* < 0.001).

Due to the key role of *caspase 3* and *caspase 9* in apoptosis, *cyclin D* and *cyclin E* in the cell cycle assay, and *MMP-2*, and *MMP-9* in the migration, the expression patterns were investigated in the treated cancerous cells. Figure 5 shows the changes in expression of *caspase 3*, *caspase 9*, *cyclin D*, *cyclin E*, *MMP-2* and *MMP-9* in the presence of Cyclo, Nio-Cyclo and Nio-Cyclo-PEG, respectively. *Caspase* genes are protease enzymes that involved in regulating programmed cell death. Studies showed that *Caspases 3* and *9* are both involved in the apoptosis pathway and have been reported to act as the initiators and executors, respectively [54,55]. Therefore, by activating the *Caspase* gene, a cascade of destructive pathways is launched and the cell death with the least effect on lateral cells occurs [54,56]. According to our results, Nio-Cyclo significantly increases *Caspase 3 and 9* expressions as compared to the free drug Cyclo (*p* < 0.001). However, the highest increase in the expression of *Caspase 3* and *9* genes was reported for the drug loaded nanocarrier, i.e., Nio-Cyclo-PEG (*p* < 0.001) (Figure 5A,B). As such, Nio-Cyclo-PEG can be a strong death stimulant for gastric cancer cells via apoptosis [56,57]. The results also showed a significant reduction in the expression of *Cyclin D*, *Cyclin E*, *MMP-2* and *MMP-9* genes in the presence of Nio-Cyclo as compared to Cyclo (*p* < 0.001). In AGS cells, the greatest reduction in the expression was observed for the Nio-Cyclo-PEG (*p* < 0.001) (Figure 5C–F). Therefore, Nio-Cyclo-PEG formulation can play a key role in the healing process of cancer treatment. Cyclins are proteins that activate cyclin-dependent kinases and thus regulate the cell progression through the cell cycle. Studies have shown that there is a relationship between the *cyclin* overexpression and gastric cancer [58]. Among the cyclin family, isoform D1 has been shown to be significantly associated with gastric cancer, and its overexpression leads to cancerous responses and weakness in patients [59]. Studies have also shown that this overexpression is necessary because it regulates the onset of replication at the end of G1 [60]. In addition, studies have shown that the overexpression of *MMP-2* and *MMP-9* is an important factor in the destruction of the external matrix of cancer cells. With the destruction of the extracellular matrix of cancer cells, cell connections are reduced, and the possibility of migration and metastasis is increased [61,62].

### 2.8. Apoptosis Analysis

**Figure 6:**
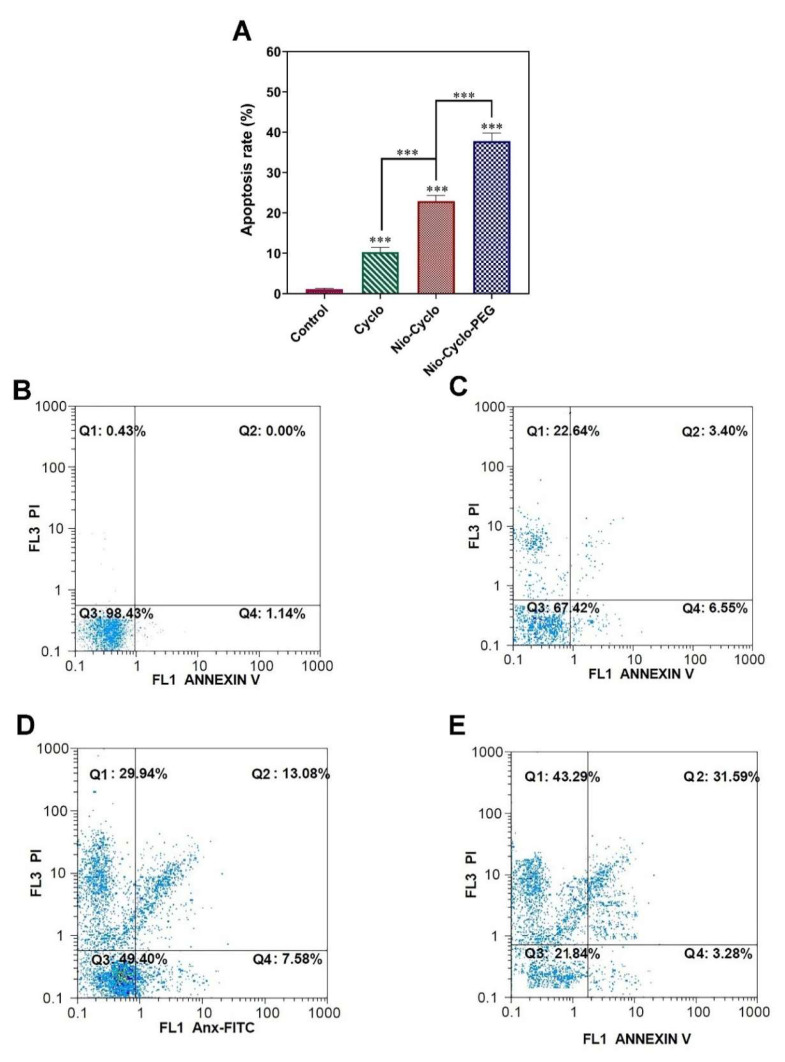
(**A**) The quantitative apoptosis rate analysis of AGS cancer cells after the treatment with different drug samples. The flow cytometry of AGS cells after treatment with (**B**) control (no drug), (**C**) Cyclo (free drug), (**D**) Nio-Cyclo and (**E**) Nio-Cyclo-PEG, where Q1: percentage of necrotic cells; Q2: percentage of late apoptotic cells, Q3: percentage of live cells and Q4: percentage of early apoptotic cells. All data are represented as Mean ± SD from three independent experiments ***: *p* < 0.001).

The apoptosis rates (%) of the AGS cells after treatment with Cyclo, Nio-Cyclo, Nio-Cyclo-PEG, and control were 10.28, 22.95, 37.81, and 1.14, respectively (Figure 6A). This increase was significantly higher in the Nio-Cyclo-PEG than the Cyclo and Nio-Cyclo formulations. These data confirmed that nanocarriers promote active-targeting drug delivery to the cancer cells. The results in Figure 6B–E show the effects of the different formulations on gastric cancer cells. In this figure, Q1, Q2, Q3, and Q4 represent necrosis, primary apoptosis, viable cell, and secondary apoptosis, respectively. In addition, the flow cytometry results show that the highest rate of apoptosis occurred in the presence of Nio-Cyclo-PEG (31.59% for primary apoptosis and 3.28% for secondary apoptosis. The as-obtained apoptosis rates (Q2 + Q4) for Cyclo, Nio-Cyclo, Nio-Cyclo-PEG, and control were 9.95%, 20.66%, 34.87%, and 1.14%, respectively. As shown in Figure 6B–E, the increased apoptosis rate is associated with a decrease in the number of living cells (Q3). It should be noted that the rate of apoptosis varies with the rate of cell necrosis. Apoptosis is a type of programmed cell death that is controlled by activating the intracellular signal pathways. It can also activate the immune system. On the other hand, necrosis can be induced by external factors [63,64]. Figure 6B–E show that the rate of necrosis (Q1) also increases in the presence of Cyclo, Nio-Cyclo, and Nio-Cyclo-PEG. Overall, gene expression analysis in this study indicates the activation of intracellular signalling pathways under the influence of drug nanocarriers like Nio-Cyclo-PEG.

### 2.9. Cell Cycle

**Figure 7:**
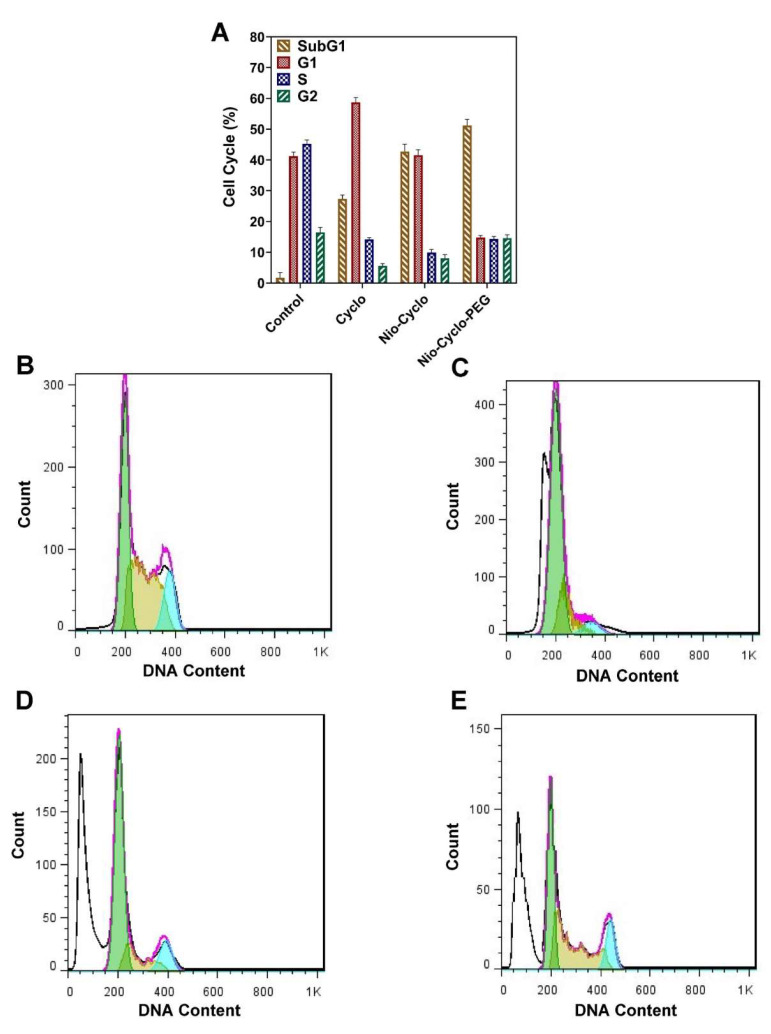
(**A**) Cell cycle analysis of different samples for AGS cancer cells. The AGS cell cycle analysis of different samples: (**B**) control, (**C**) Cyclo, (**D**) Nio-Cyclo, and (**E**) Nio-Cyclo-PEG. Data are represented as Mean ± SD from three independent experiments.

The results presented in Figure 7A revealed that the highest DNA content was detected in the presence of the Nio-Cyclo-PEG formulation. This indicates that the subG1 phase is associated with the presence of cells in the apoptosis phase. It was observed that the Nio-Cyclo-PEG formulation could enhance the rate of apoptosis much more than the Cyclo and Nio-Cyclo formulations. As can be seen, the number of cells in the subG1 phase increases with the placement of drug in the niosomes, and finally this value reaches its maximum with PEGylation. The results for the cell cycle are given in Figure 7B–E.

### 2.10. Scratch Assay

**Figure 8:**
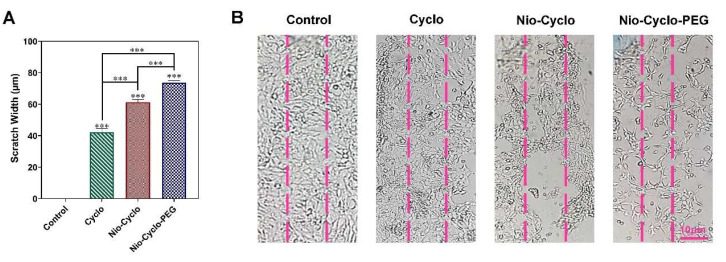
(**A**) The inhibitory effects (scratch widths of samples) of different drug formulations on the migration of the AGS after 72 h of treatment; (**B**) Light microscopy images of AGs cells after treatment for 72 h. The inhibitory effects of different drug formulations, including control (no drug), Cyclo, Nio-Cyclo, and Nio-Cyclo-PEG (from left to right) on migration of the gastric cancer cells; Data are represented as Mean ± SD from three independent experiments ***: *p* < 0.001).

The effect of each drug formulation on cell metastasis was investigated using the wound scratch method. According to Figure 8A, the migration of AGS cells in the presence of Cyclo and Nio-Cyclo and Nio-Cyclo-PEG samples decreased. The measured widths were 42.37, 61.29, and 73.64 nm for the Cyclo, Nio-Cyclo, and Nio-Cyclo-PEG, respectively. The comparison of results in Figure 8B showed that the Nio-Cyclo-PEG exhibited the most inhibitory effect on the cell migration among other tested samples.

## 3. Conclusions

In this study, PEGylated niosomes loaded with cyclophosphamide were successfully designed for pH-responsive drug delivery to gastric cancer cells. The niosomal nanocarrier formulations were comprehensively investigated and optimized in terms of their size, PDI, and drug loading by the response surface methodology. Based on the results, the optimal formulation for niosomal nanocarriers showed controlled drug release with improved stability over the studied storage time. Moreover, the release profile of drug was improved in the pathological cancerous environment, where the acidic pH of cancer tissue preferentially triggering the drug release, thereby accelerating the treatment process. Additionally, cell viability studies show that the cancer cells treated with Nio-Cyclo-PEG exhibited a significant reduction in number compared with other tested formulations. It also caused apoptosis in cancer cells by regulating the expression of Caspase 3, Caspase 9, Cyclin D, Cyclin E, MMP-2 and MMP-9 genes. Interestingly, the PEGylated formulation was found to have improved cytotoxicity against cancer cells. This result shows that the Nio-Cyclo-PEG drug formulation is effective even at low dosage, leading to lower systemic toxicity when administered to the patient. In addition, these drug-loaded nanocarriers reduced the migration of cancer cells, which is advantageous for preventing the metastasis. In conclusion, we have developed an effective drug-delivery nanocarrier for the targeted treatment of gastric cancer that has minimal effects on non-cancerous tissues.

## 4. Experimental Section

### 4.1. Chemicals

Cyclophosphamide (C_7_H_15_Cl_2_N_2_O_2_P, purity >99%) was obtained from BAXTER Company (Illinois, United States). Span 60 (sorbitan monostearate, C_24_H_46_O_6_, purity >99%), Span 20 (Sorbitan monolaurate, C_18_H_34_O_6_, purity >99%), and cholesterol were bought from Sigma-Aldrich Chemical Co. (St. Louis, USA). Chloroform (CHCl_3_, purity >99.9%), polyethylene glycol 2000 (PEG 2000, H(OCH_2_CH_2_)Noh, purity >99.9%), dimethyl sulfoxide (DMSO, (CH_3_)_2_SO, purity >98%), dialysis membrane (MWCO 12000 Da), phosphate-buffered saline (PBS), sodium dodecyl sulfate (SDS, NaC_12_H_25_SO_4_, purity >99%), Amicon (Ultra-15-Membrane, MWCO 30000 Da), and methanol (CH_3_OH, purity >99%) were supplied from Merck Chemical Co. (Darmstadt, Germany). The AGS cell line (C131) and HFF cell line (C163) were obtained from Pasteur Institute of Iran. Dulbecco’s modified Eagle’s medium (DMEM, purity >99.9%), trypan blue (C_34_H_28_N_6_O_14_S_4_, purity >99.9%), trypsin-EDTA (purity >99%), RPMI-1640, MTT (3-(4,5-Dimethylthiazol-2-yl)-2,5-diphenyltetrazolium bromide, purity >99.9%), and Penicillin/Streptomycin (PS, C_39_H_61_N_9_O_16_S, purity >99%) 100X and fetal bovine serum (FBS, purity >99%) were procured from Gibco (Life Technologies). Annexin V/propidium iodide (PI) (Apoptosis detection kit) was received from Roche (Germany). RNA extraction kit was purchased from Qiagen (USA). Revert AidTM First Strand cDNA Synthesis Kit (Fermentas, Vilnius, Lithuania) was used to synthesize the cDNA. The other reagents and solutions were analytical grade. MilliQ water was used to prepare the aqueous solutions.

### 4.2. Optimization of Niosomal Formulations Using Response Surface Methodology

Response surface methodology (RSM) was used for the optimization of the niosomal formulations. In this study, central composite design (CCD) with three levels for two numerical variables and one categorical variable was used to optimize different formulations. Two numerical factors (surfactant: cholesterol molar ratio and lipid: drug molar ratio) and one categorical factor (type of surfactant) were specified to investigate their impacts on niosomal particle size (nm), polydispersity index (PDI), and entrapment efficiency percentage (EE%). The nonlinear quadratic equation for the responses was generated using the Design Expert software (Version 10, State-Ease Inc., Minneapolis, MN, USA). The surfactant type and the molar ratio ranges of independent variables along with their low, medium and high levels are shown in Tables S1 and S2. The formulation with the smallest particle size, narrow polydispersity index. and high entrapment efficiency (%) was considered the best formulation and used for further investigations.

### 4.3. Synthesis of Cyclophosphamide-Loaded Niosomes and PEGylated-Nio-Cyclo

Cyclophosphamide-loaded niosomes (Nio-Cyclo) were prepared using thin-film hydration as reported previously with small modifications [65]. Briefly, molar ratios of Span20:Span60 and cholesterol (0.5, 1 and 2) were dissolved in 9 mL of chloroform/methanol (2:1; *v*/*v*) (as organic solvent) containing 10 mg of drug, and were mixed in a 50 mL round bottom flask. The solvents were evaporated in a round-bottom flask under constant rotation in a rotary evaporator (Totary evaporator, Heidolph Instrument, Schwalbach, Germany) at 60 °C and 150 rpm to form a thin lipid film. Then, the dried thin films were hydrated using 1X PBS concentration (10 mL, pH 7.4) at 60 °C (150 rpm, 30 min). Finally, the samples were sonicated for 7 min using a probe sonicator (UP50H compact laboratory homogenizer, Hielscher Ultrasonic, Teltow, Germany) to achieve the cyclophosphamide-loaded niosomes with a uniform size distribution. The samples were stored in a refrigerator (4 °C) for further analysis. The optimized PEGylated niosome was made by adding 0.5 mg PEG 2000 and placed in a sonicator for 15 min (Scheme 1). Finally, it was homogenized for 10 min at 12,000 rpm. The combination of different niosomal formulations can be found in Table 1.

### 4.4. Characterizations of Niosomal Formulation

The particle size, polydispersity index (PDI), and zeta potential were measured using a zeta sizer (Malvern Panalytical, Malvern, UK) at 25 °C. A scanning electron microscope (NOVA NANOSEM 450, FEI Company, Hillsboro, OR, USA) was utilized to analyse the morphologies of the prepared niosomes. A diluted sample (1:100) was located on the SEM holder and coated with a layer of 100 Å gold for 3 min under argon at a pressure of 0.2 atm. Additionally, the best niosomal formulation’s shape and morphology were analysed with transmission electron microscopy (TEM). For this, a small amount of sample was placed on a carbon film-covered copper grid and stained with a 1% phosphotungstic acid. Finally, the sample was imaged using TEM at 100 kV (Zeiss EM900 Transmission Electron Microscope, Carl Zeiss AG, Oberkochen, Germany) [66]. The FTIR spectra of cyclophosphamide, Span 60, PEG 2000, cholesterol, cyclophosphamide-loaded niosome (Nio-Cyclo), and PEGylated cyclophosphamide-loaded niosome (Nio-Cyclo-PEG) were investigated in KBr discs using a PerkinElmer FTIR spectrophotometer (spectrum Two, USA). FTIR analysis were conducted in a scanning range of 4000 to 400 cm^−1^ in a fixed resolution of 4 cm^−1^ at room temperature.

### 4.5. Analysis of Entrapment Efficiency

Entrapment efficiency was measured indirectly by quantifying free cyclophosphamide after centrifugation (Eppendorf^®^ 580R centrifuge, Germany) of best niosomal formulation with an Amicon Ultra-15-membrane at 4000 rpm for 20 min at 4°C. The drug-containing niosomes were maintained in the upper chamber during filtration, and free drugs moved through the membrane. The free drug concentration was then measured at a wavelength of 210 nm using UV-Vis spectrophotometer (Shimadzu UV-1700 Pharma spec, Japan). The percentage of entrapment efficiency was calculated using the following equation [67].

(1) $$\mathrm{Entrapment}\;\mathrm{efficiency}\;\%=\;\left[\left(A-B\right)/A\right]\times 100$$

where *A* is the initial cyclophosphamide concentration for niosomal preparation and *B* is the concentration of unentrapped cyclophosphamide after centrifugation.

### 4.6. In Vitro Drug Release Study

Two millilitres of Nio-Cyclo and Nio-Cyclo-PEG was mixed in a semipermeable-acetate cellulose membrane dialysis bag and then immersed in 50 mL release medium containing PBS-SDS (0.5% *w*/*v*) under slow stirring (50 rpm) at 37 °C for 72 h in three different pHs (7.4, 5.4 and 1.2). At specific time intervals (1, 2, 4, 8, 24, 48, and 72 h), 1 mL of released medium was sampled and replaced with an equal volume of fresh release medium to maintain the sink condition [68]. The released quantity was measured at 210 nm using UV-visible spectroscopy. The test was also conducted for free drug as control, where the drug concentration was equivalent in dialysis bag.

### 4.7. Release Kinetic Modelling

The release data were linearly fitted (linear regression (*R*) analysis) to the different mathematical models including Korsmeyer–Peppas (log cumulative % drug release vs. log time), Higuchi (cumulative % drug release vs. square root of time), first-order (cumulative % drug remaining vs. time), and zero order (cumulative % drug release vs. time) to determine the release kinetic [44]. The zero-order model is associated with drug dissolution, which is concentration-independent. The first-order model describes the drug release in a concentration-dependent manner [44]. The simplified Higuchi and Korsmeyer–Peppas models define the release of drug from the matrix and polymeric systems [69]. The initial 60% release is ordinarily sufficient to ascertain the most suited model for drug release [70].

### 4.8. Stability Study

Nio-Cyclo and Nio-Cyclo-PEG were kept in two different conditions (25 ± 1 °C and 4 ± 1 °C/60% RH ± 5% RH) for two months, and the changes in particle size, PDI, and amount of drug in niosomal formulations were evaluated at specific time intervals (14, 30, and 60 days).

### 4.9. Cytotoxicity Study

The proportion of living human foreskin skin fibroblasts (HFF) and AGS cells after treatment with niosomal formulations was evaluated by MTT assay. The cells were seeded at a density of 1 × 10^4^ cells/well in RPMI-1640 supplemented with 1x penicillin-streptomycin and 10% FBS. After 24 h incubation, the cells were treated with different concentrations of Cyclo, Nio-Cyclo, and Nio-Cyclo-PEG (0, 15.63, 31.25, 62.5, 125, and 250 μg/mL). After 48 and 72 h, 20 μL MTT (5 mg/mL) was added to each well and the plates were then incubated for 4 h at 37 °C under 5% CO_2_. The viability of cells was calculated by measuring the absorbance at a wavelength of 570 nm using ELISA micro-reader (Organon Teknika, Oss, The Netherlands) and following equation [71].

Cell viability % = (OD_570_ sample/OD_570_ control) × 100 (2)

### 4.10. Apoptotic Gene Expression Analysis

In order to evaluate the expression of apoptosis-related genes including *MMP-2*, *MMP-9*, *caspase 3*, *caspase 9*, *cyclin D* and *cyclin E*, and *ß-actin* (as internal control), total RNA was extracted from the treated cells according to the kit instructions (RNA extraction kit, Qiagen, Germantown, MD). cDNA synthesis Kit (Fermentas, Vilnius, Lithuania) was used to create cDNA from RNAs. The mixture contained buffer (5 μL, 5X), total RNA (1 μg), random hexamer primer (0.5 μL), oligo dT primer (0.5 μL), deoxynucleotide triphosphate mixture (2 μL, 10 mM), RNase enzyme inhibitor (1 μL, 20 units/microliter), opposite transcriptase enzyme (1 μL), and double-distilled water (up to a final volume of 20 μL). The PCR program was set as follows; 25 °C (5 min), 42 °C (60 min), 70 °C (5 min), and 4 °C (5 min). The primers used for the studied genes are listed in Table S3. The last cycle was applied to conduct the real-time PCR reaction as follow, 95 °C (1 min) 95 °C for 15 s and 60 °C (1 min). All analysis were performed based on IC50 values obtained from the previous step. Finally, Delta-Delta Ct (2^−ΔΔCT^) method was used to calculate the fold change expression of genes.

### 4.11. Apoptosis Analysis

To check apoptosis, double-staining with Annexin V-FITC/PI (Roche, Mannheim, Germany) was used. In this method, AGS cells were treated with IC50 concentration of Cyclo, Nio-Cyclo and Nio-Cyclo-PEG for 72 h at 37 °C. Then, cells were detached using Trypsin-EDTA, washed with PBS, and centrifuged for 5 min at 1500 rpm. The resulting cell mass was resuspended in 500 μL binding buffer and stained with 3 μL Annexin V-FITC for 5 min in a dark condition. The cells were then washed with PBS and after adding the binding buffer, they were stained with 5 μL propidium iodide (PI) solution [63,64]. Cells without receiving drug treatment served as control. Eventually, the samples were examined using flow cytometry in 488 nm and the data were processed using FlowJoTM software (Tree Star, Ashland, OR, USA).

### 4.12. Cell Cycle

Propidium iodide (PI) staining was used to assess the cell cycle. In this method, the genomic DNA content of cells is attached to PI and allows the identification of the cell cycle stage. The cells were seeded in 6-well plates at a density of 1 × 10^6^ cells/well and incubated overnight. The cells were washed 3 times with PBS and then placed in complete medium for 72 h. After incubation, the cells were exposed to 70% cold ethanol overnight in 4 sets and stained with 500 μL PI solution (containing RNase) for 20 min at room temperature in dark. Finally, the samples were evaluated using flow cytometry. All experiments were performed on IC50 concentration in triplicate.

### 4.13. Scratch Assay

The anti-metastatic property of each formulation was evaluated using scratch method. After culturing the cells in a 6-well plate, they are gently scratched by a 200 μL pipette tip. The cells were then washed twice with PBS and incubated with 5% CO_2_ at 37 °C by adding fresh culture medium. Finally, the plate was evaluated with a fluorescence microscope (Nikon Eclipse Ti, Nikon Instruments Inc, Melville, NY, USA) to determine the rate of cell migration.

### 4.14. Statistical Analysis and Curve Fitting

Statistical analysis and curve fitting were carried out using GraphPad Prism software (version 8, Dotmatics Inc., Boston, MA, USA). All data were reported as mean ± SD from three independent experiments. One-way and two-way analysis of variance (ANOVA) were used to measure the significance level (*p* value < 0.05) of difference between groups. Central composite design (CCD) was performed using Design-Expert software version 10 (Stat-Ease Inc., Minneapolis, MN, USA).

## Figures and Tables

**Table 1:** Design of the experiment based on response surface methodology for the optimization of cyclophosphamide-loaded niosomes and the corresponding results.

Run	Levels of Independent Variables			Dependent Variables			
	Lipid: Drug (mol Ratio)	Surfactant: Cholesterol (mol Ratio)	Surfactant Type	Average Particle Size (nm)	Polydispersity Index	Entrapment Efficiency (%)	Loading Capacity (%)
1	−1	1	Span60	214.8	0.184	88.75	5.24
2	0	0	Span60	155.3	0.162	92.35	2.86
3	0	0	Span60	168.4	0.189	93.79	2.36
4	1	0	Span20	239.8	0.275	84.45	1.96
5	−1	−1	Span20	286.4	0.287	75.42	4.93
6	0	−1	Span20	294.1	0.346	81.08	2.74
7	1	1	Span20	275.3	0.293	92.75	2.19
8	0	0	Span20	193.4	0.189	82.19	2.83
9	0	−1	Span60	249.5	0.255	83.34	2.63
10	1	−1	Span20	326.1	0.329	79.92	1.82
11	1	−1	Span60	243.4	0.346	82.43	1.75
12	0	0	Span20	198.2	0.205	79.78	2.74
13	−1	0	Span60	162.1	0.231	83.79	5.03
14	1	0	Span60	224.2	0.218	95.83	2.00
15	1	1	Span60	286.4	0.315	97.74	2.00
16	0	0	Span60	173.5	0.193	95.14	2.95
17	−1	0	Span20	185.7	0.285	84.61	5.53
18	0	1	Span20	258.6	0.341	85.70	3.00
19	0	0	Span20	192.1	0.211	77.24	2.66
20	0	1	Span60	230.7	0.196	86.49	3.99
21	−1	1	Span20	232.5	0.194	84.63	5.73
22	−1	−1	Span60	279.3	0.307	81.92	5.01

**Table 2:** Analysis of variance of entrapment efficiency (EE), particle size, and PDI for quadratic polynomial model.

Source	*f*-Value	*p*-Value Prob > *f*
**Particle Size (nm)**		
Model	20.00	<0.0001
A	16.22	0.0014
B	9.62	0.0084
C	11.17	0.0053
AB	5.42	0.0368
AC	0.017	0.8981
BC	2.95	0.1098
A^2	4.57	0.0521
B^2	91.05	<0.0001
**PDI**		
Model	3.57	0.0206
A	3.63	0.0792
B	5.27	0.0390
C	3.07	0.1031
AB	1.46	0.2490
AC	0.030	0.8661
BC	0.27	0.6101
A^2	2.00	0.1812
B^2	9.46	0.0089
**EE (%)**		
Model	4.90	0.0058
A	6.11	0.0280
B	14.27	0.0023
C	15.70	0.0016
AB	1.16	0.3009
AC	0.44	0.5207
BC	9.921 × 10^−3^	0.9222
A^2	0.25	0.6262
B^2	1.47	0.2472

**Table 3:** Regression analysis results for particle entrapment efficiency, particle size, and polydispersity index (PDI) for the quadratic model.

Models
**Particle Size (Span20) =** +194.86 + 20.17 × A − 23.37 × B + 13.83 × A × B + 15.96 × A2 + 71.23 × B2 **Particle Size (Span60) =** +170.91 + 18.90 × A − 6.72 × B +13.82 × A × B + 15.96 × A2+ 71.23 × B2
**PDI (Span20) =** +0.221 + 0.022 × A − 0.022 × B + 0.019 × A × B + 0.027 × A2 + 0.060 × B2 **PDI (Span60) =** +0.189 + 0.026 × A − 0.035 × B + 0.019 × A × B + 0.027 × A2 + 0.060 × B2
**EE (Span20) =** +83.21 + 2.08 × A + 4.44 × B + 1.51 × A × B + 0.88 × A2 − 2.14 × B2 **EE (Span60) =** +89.92 + 3.59 × A + 4.22 × B + 1.51 × A × B +0.88 × A2 − 2.14 × B2

**Table 4:** Results of regression analysis for three responses.

Response	R-Squared	Adj R-Squared	Adeq Precision	Lack of Fit
Particle size	0.9249	0.8786	13.378	0.0314
PDI	0.6872	0.4946	5.201	0.0128
EE (%)	0.7509	0.5976	8.407	0.0637

**Table 5:** The optimized values for the three responses obtained by RSM and the experimental responses under optimum condition.

Parameter	Predicted by RSM	Experimental Data (Nio-Cyclo)	Nio-Cyclo-PEG	Niosome (Nio)
Average size (nm)	171.656	188.100 ± 9.200	170.600 ± 5.530	145.540 ± 6.200
polydispersity index (PDI)	0.179	0.170 ± 0.014	0.145 ± 0.007	0.191 ± 0.012
Entrapment efficiency (EE) (%)	90.337	92.580 ± 1.120	94.870 ± 0.720	-

**Table 6:** The kinetic release models and their parameters for different niosomal formulations.

Kinetic Model		Zero-Order	First-Order	Higuchi	Korsmeyer–Peppas	
		Ct = C0 + K0t	LogC = LogC0 + Kt/2.303	Q = KH√t	Mt/Mꝏ = Kt.tn	
		r2	r2	r2	r2	n *
Free Cyclophosphamide (Cyclo)	pH 7.4	0.4854	0.9305	0.6586	0.7666	0.5135
Nio-Cyclo	pH 7.4	0.8508	0.9133	0.9599	0.9679	0.5384
	pH 5.4	0.8306	0.9467	0.9468	0.9556	0.4829
	pH 1.2	0.6929	0.9588	0.8476	0.9058	0.3996
Nio-Cyclo-PEG	pH 7.4	0.8609	0.9082	0.9639	0.9730	0.6304
	pH 5.4	0.8644	0.9393	0.9666	0.9682	0.5519
	pH 1.2	0.7809	0.8991	0.9133	0.9353	0.4288

* Diffusion or release exponent.

## Supplementary Materials

The following supporting information can be downloaded at: https://www.mdpi.com/article/10.3390/molecules27175418/s1, Figure S1: Zeta potential for samples; Figure S2: Stability data at different temperatures; Table S1: Different levels of independent variables used by Central Composite design; Table S2: Two types of categorical variables used by Central Composite design, Table S3: The primers used in PCR.
