# Supplementary material for: pH-Responsive PEGylated Niosomal Nanoparticles as an Active-Targeting Cyclophosphamide Delivery System for Gastric Cancer Therapy
Source: Molecules. 2022 Aug 24;27(17):5418. doi: 10.3390/molecules27175418 (PMC9457647; doi:10.3390/molecules27175418)
Supplement: Supplementary file 1 [file molecules-27-05418-s001.zip › molecules-1819346-supplementary.pdf]

Supporting Information

pH-Responsive PEGylated Niosomal Nanoparticles as an Active-Targeting Cyclophosphamide Delivery System for Gastric Cancer Therapy

Farnaz Khodabakhsh [1,†,*], Mahsa Bourbour [2,†], Mohammad Tavakkoli Yaraki [3], Saina Bazzazan[4], Haleh Bakhshandeh[5], Reza Ahangari Cohan[5,*], Yen Nee Tan[6,7,*]

1 Department of Genetics and Advanced Medical Technology, Medical Biotechnology Research Center, Faculty of Medicine, AJA University of Medical Sciences, Tehran 1411718541, Iran
2 Department of Biotechnology, Alzahra University, Tehran 1993893973, Iran
3 School of Natural Sciences, Macquarie University, Sydney, NSW 2109, Australia
4 Department of Community Medicine, Mashhad Branch, Islamic Azad University, Mashhad 9187147578, Iran
5 Department of Nanobiotechnology, New Technologies Research Group, Pasteur Institute of Iran, Tehran 1316943551, Iran
6 Faculty of Science, Agriculture and Engineering, Newcastle University, Newcastle Upon Tyne NE1 7RU, UK
7 Newcastle Research and Innovation Institute, Newcastle University in Singapore, Singapore 609607, Singapore
* Correspondence: farnaz.khodabakhsh@ajaums.ac.ir (F.K.); cohan_r@yahoo.com (R.A.C.); Yennee.tan@newcastle.ac.uk (Y.N.T.)
† These authors contributed equally to this work.

Table S1. Different levels of independent variables used by Central Composite design.

Level	-1	0	+1
A (Lipid: Drug, molar ratio)	10	20	30
B (Surfactant: Cholesterol, molar ratio)	0.5	1	2

Table S2. Two types of categorical variables used by Central Composite design.

Factor	Name	Type	Level 1	Level 2
C	Surfactant type	Categoric	Span20	Span60

Table S3. The primers used in PCR

Gene	Primer Sequence
Cyclin D	Forward: 5'- CAGATCATCCGCAAACACGC-3′<br>Reverse: 5'- AAGTTGTTGGGGCTCCTCAG-3'
ß-actin	Forward: 5’- TCCTCCTGAGCGCAAGTAC-3’<br>Reverse: 5’- CCTGCTTGCTGATCCACATCT-3’
Caspase 3	Forward: 5'- CATACTCCACAGCACCTGGTTA-3′<br>Reverse: 5'- ACTCAAATTCTGTTGCCACCTT-3'
Caspase 9	Forward: 5’-CATATGATCGAGGACATCCAG-3<br>Reverse: 5’-TTAGTTCGCAGAAACGAAGC-3’
Cyclin E	Forward: 5'- CTCCAGGAAGAGGAAGGCAA-3′<br>Reverse: 5'- TTGGGTAAACCCGGTCATCA-3'
MMP-2	Forward: 5'- TTG ACG GTA AGG ACGGAC TC -3′<br>Reverse: 5'- CAT ACT TCA CAC GGA CCA CTTG -3'
MMP-9	Forward: 5'- GCACGACGTCTTCCAGTACC -3′<br>Reverse: 5'- CAGGATGTCATAGGTCACGTAGC -3'

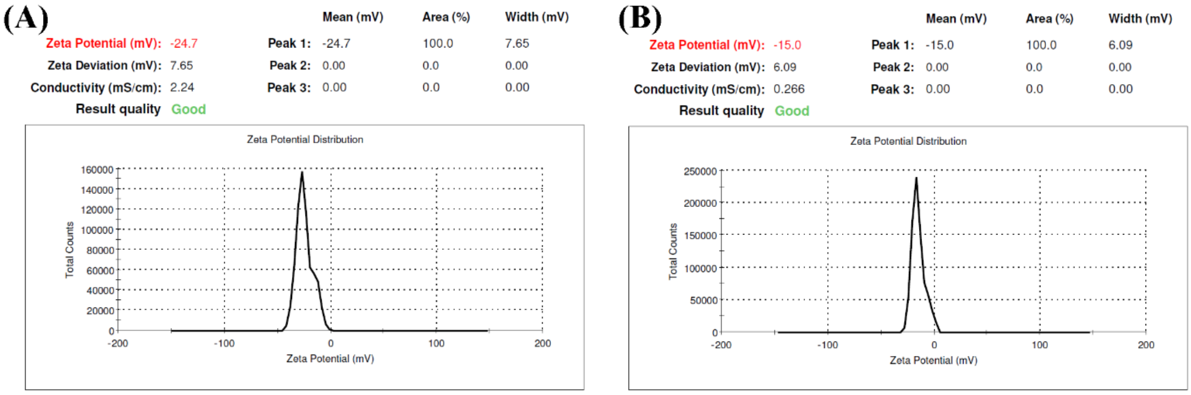

Figure S1. Zeta potential measurement of Nio-Cyclo (A), and Nio-Cyclo-PEG (B).

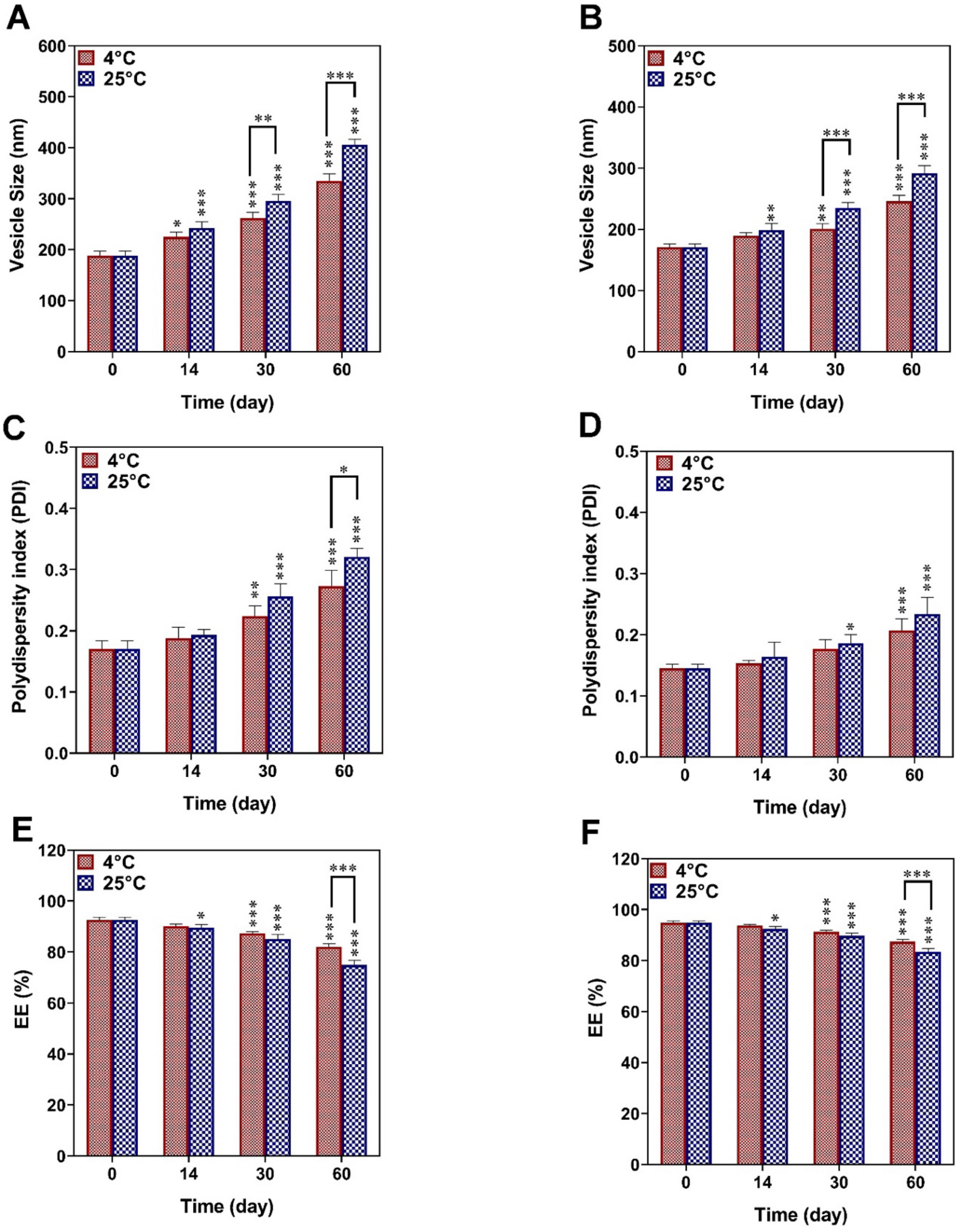

Figure S2. The impact of storage time (14, 30 and 60 days) and storage temperature (4 ˚C and 25 ˚C) on a, b) The average size, c, d) the polydispersity index (PDI), and e, f) The entrapment efficiency of cyclophosphamide in Nio-Cyclo and Nio-Cyclo-PEG formulations (Data presented as average ± SD); The P-values are *: $P<0.05$, **: $P<0.01$ and ***: $P<0.001$.
